# Patients’ and parents’ percetion of care on a paediatric interprofessional training ward

**DOI:** 10.1186/s12909-019-1813-6

**Published:** 2019-10-16

**Authors:** Christine Straub, Sebastian F. N. Bode

**Affiliations:** Center for Pediatrics – Department of general pediatrics, adolescent medicine, and neonatology, Medical Center – University of Freiburg, Freiburg im Breisgau, Germany

**Keywords:** Interprofessional training ward, Patients’ evauation, Parental evaluation, Medical education, Interprofessional learning, Interprofessional collaboration

## Abstract

**Background:**

Interprofessional training wards (ITWs) have been established in different fields of adult medicine to promote interprofessional learning and interprofessional collaboration of health care profession students. High patient satisfaction rates have been reported for ITWs. No data of parents’ and especially patients’ evaluation of care on a paediatric ITW have been reported so far. This study aims to evaluate parents’ and patients’ perceptions of medical and nursing care on a paediatric ITW.

**Methods:**

In 2017 we established and started an interprofessional training ward in the setting of a general paediatric ward (IPAPAED). Medical students and nurse trainees care for 4–6 patients under supervision of registered nurses and certified physicians. All parents and all patients older than 8 years were invited to evaluate different aspect of their care on the IPAPAED.

**Results:**

Since November 2017 until February 2019 parents (*n* = 109) rated the overall care of their children on the IPAPAED ward with m = 1.21 (SD ± .43) (1 = “excellent”, 4 = “poor”). Patients (*n* = 56) rated their overall care with m = 1.29 (SD ± 0.5). Other aspects of care and interprofessional collaboration were rated equally well. Analysis of the (limited) free-text commentaries revealed that perceived quality of care, friendliness and communication were especially valued by patients and parents.

**Discussion & conclusion:**

On a paediatric ITW, in the view of parents and patients in our sample, a high level of care is delivered and satisfaction rates are excellent. An ITW seems, from a patient and parent point of view, feasible, even in paediatrics.

## Background

Challenges in today’s complex healthcare should be dealt with by an interprofessional team to ensure optimal patient outcomes [1]. Clinical education wards (CEWs) are educational interventions to promote active learning of students of different health care professions from, with, and about each other by involving them directly in patient care and allowing them to take on responsibility for the care of patients under supervision by experienced health-care professionals to prepare them for their future working together as an interprofessional team [2, 3]. There were fears that this handing over of responsibility could detrimentally affect care [3]. The feasibility of students taking on responsibility for patient care has been demonstrated extensively as have positive effects on professional role development, interprofessional competencies, patient outcomes and cost effectiveness [3–14].

Also concerns that allowing students to care nearly independently for patients might not be accepted by patients have been shown to be unfounded as both monoprofessional CEWs for medical students have reported positive patient perceptions [3, 12] as have interprofessional training wards (ITWs) [4–10]. In some cases patient satisfaction was even higher on ITW wards compared to conventional wards [5, 8]. Students’ empathy, understandable communication, personal care, enthusiasm, and motivation were especially commended by patients [3–10]. On ITWs, medical students and nursing students, sometimes also other allied health professional students, work and learn together and about each other [1].

Reported ITWs were mainly established in the setting of orthopaedic wards as well as in other areas of adult medicine [4–10]. Data on parental evaluation exist for a paediatric monoprofessional CEW [3]. Overall satisfaction was high and no differences in medical treatment success, complications rates, and overall care could be identified when compared to a control group in the same, as well as other hospitals [3]. This supports the concept that medical students can, under appropriate supervision, take care of paediatric patients with no negative effects on both quality of care and parents’ perception of care.

To our knowledge no patient satisfaction and evaluation data have been reported from paediatric ITWs so far. Neither the paediatric ITW that existed in Stockholm, nor the paediatric interprofessional emergency department at Karolinska Institutet in Stockholm, Sweden, have published data, nor are patients/parent evaluation data reported from an ITW-like setting that existed in Ireland [15]. Therefore there is a need to investigate how care on a paediatric ITW is perceived by patients and their families if they are cared for primarily by nurses and physicians in training. The paediatric setting poses special challenges in general, but especially for participants, including age-appropriate communication with both patients and parents, a wide age diversity, and a plethora of different clinical pictures.

**Fig. 1:**
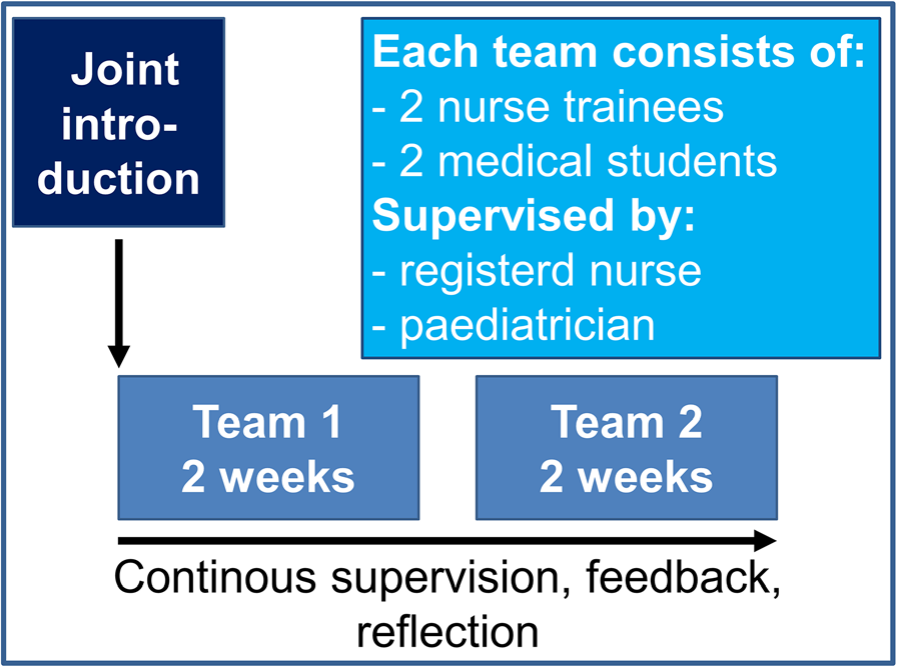
IPAPAED ward setup

In November 2017 we established an interprofessional education ward in the setting of a general paediatric ward (IPAPAED). Here, medical students and nurse trainees work together for 2 weeks in teams of two medical students and two nurse trainees, both in their final year. As interprofessional teams they take on responsibility for the care of patients from the end of the first year until18 years of age. The IPAPAED is integrated in a general paediatric ward and the team takes care of four to six patients. Nurses and physicians in training are supervised by registered nurses and certified medical doctors as described elsewhere [16]. As nurse trainees alter between practical and theoretical “education blocks” of 4 weeks, the IPAPAED ITW takes place non-continuously for “blocks” of 4 weeks each (Fig. 1). Evaluation results are reported for the blocks in November 2017, February 2018, May/June 2018, October/November 2018, and February 2019. In between the “blocks” nurse trainees in the first 2 years of their course, and medical students in their last year are routinely involved in patient care but they do not receive interprofessional preparation and supervision.

All participants of the IPAPAED are specifically prepared for interprofessional teamwork, communication, feedback, and ward specific activities in advance. Special emphasis is put on patient-centered care and participants are instructed to include patients and their families as far as possible in decision making and care planning while on the IPAPAED ward. The ITW rotation on the IPAPAED is the first chance for all participants to actually be the first professional contact persons for patients and their families during their hospital stay. Specific aims were to prepare the nurses and physicians in training for their later role as members of interprofessional teams. Participants are supervised by experienced nurses and physicians who receive specific training on interprofessional teaching. They ensure that both nurses and physicians in training bring their expertise into patient care, supervise feedback, help with time management, and provide a safety net for participants of the IPAPAED. Supervisors use a specifically designed form to identify aspects that went well as well as areas that needed to be improved, and to identify possible errors in treatment [16].

## Methods

We developed a questionnaire regarding general aspects of care and rating of the IPAPAED ward based on an example from a monoprofessional training ward [11]. Our questionnaire comprised of the following questions:

1. Please rate the overall care you received during your hospital stay on the IPAPAED ward (1 = excellent, 2 = good, 3 = fair, 4 = poor).
2. Please rate the impact that nurse trainees had on the care you received (1 = very positive, 2 = positive, 3 = no effect, 4 = slightly negative, 5 = negative).
3. Please rate the impact that medical students had on the care you received (1 = very positive, 2 = positive, 3 = no effect, 4 = slightly negative, 5 = negative).
4. Please rate the collaboration of nurses in training and medical students (1 = excellent, 2 = good, 3 = fair, 4 = poor).
5. Did you receive all relevant information that you needed from the IPAPAED team (1 = definitely yes, 2 = yes, 3 = rather yes, 4 = rather not, 5 = not at all)?
6. If you/your child had to be treated as an inpatient again – would you agree to be cared for on the IPAPAED again (1 = definitely, 2 = yes, 3 = rather yes, 4 = rather not, 5 = not at all)?
7. Please note any positive comments that you might have.
8. Please let us know what you think that needs to be changed regarding the IPAPAED.

Two additional questions regarding the length of stay and the age of the patient were also included.

The questionnaire was handed out to all parents of patients treated on the IPAPAED ward and all patients older than 8 years from November 2017 to February 2019. An information leaflet explaining the IPAPAED ward was handed out together with the questionnaire. Questionnaires were returned anonymously by being posted in a specifically designated letter box on the ward.

### Ethics

The study was approved by the University of Freiburg ethics committee (permit no. 561/17). As asked by the ethics committee we were not allowed to record the gender and the exact age of the patients but had to provide age-spans for participants to tick in boxes as appropriate. The ethics committee stated that written consent was not needed to be treated on the IPAPAED as both medical students and nurse trainees in the past already routinely take care of patients on regular paediatric wards under supervision of experienced nurses and doctors as this is part of their respective training. The IPAPAED ward formalizes this supervision and gives participants the chance to take on responsibility for patient care but nurses and physicians are always on the ward to ensure optimal quality of care. Verbal consent to be treated by the IPAPAED team was asked of all patients or parents and all were given the opportunity to refrain from participating in the study – but none chose to do so.

### Statistical analysis

Quantitative data were analysed with Graph Pad Prism version 7.01 (GraphPad Software, La Jolla, California, USA). The Mann-Whitney-U-Test was used to identify differences in parental or patients’ evaluation or between items of the questionnaire. Quantitative content analysis was used to analyse the free-text commentaries [17].

## Results

From November 2017 until February 2019 five IPAPAED blocks of 4 weeks (each two teams for 2 weeks, Fig. 1) took place and 180 patients were cared for by a total 20 nurses in training and 20 physicians in training. Mean length of hospital stay was 2.9 (SD ± .4) days.

A total of 109 parents (response rate 60.5%) and 56 patients (31% of total patients, 55 patients (94.8%) > 8 years) participated in the study. Patients’ age can be found in Table 1.

**Table 1:** Age distribution of parents’ children and patients who participated in the evaluation

Age span	Parents’ evaluation	Patients’ evaluation
1–3 years	12	0
4–7 years	37	1
8–10 years	18	8
11–13 years	24	24
14–16 years	15	15
17–18 years	1	4
n/a	2	4

*n/a* not available

### Quantitative feedback

Parents rated the overall care their children received on the IPAPAED ward with m = 1.21 (SD ± .43). Patients rated their overall care with m = 1.29 (SD ± .5). Care by nurses and physicians in training was rated equally good by parents and patients as was perceived collaboration between professions as well as information sharing (Table 2). Two patients stated (one 11–13 years, one 17–18 years) that they “rather not” received all the information they needed. No significant differences in the evaluation of the different items by either patients or parents were found.

**Table 2:** Evaluation of different items by parents and patients

	parents	Patients
Impact of nurses in training on overall care	(*n* = 109) m = 1.35, SD ± .6	(*n* = 55) m = 1.42, SD ± .64
Impact of physicians in training on overall care	(*n* = 109) m = 1.36, SD ± .59	(*n* = 56) m = 1.41, SD ± .63
Interprofessional collaboration	(*n* = 103) m = 1.39, SD ± .49	(*n* = 56) m = 1.38, SD ± .53
Information sharing	(*n* = 109) m = 1.4, SD ± .53	(*n* = 53) m = 1.65, SD ± .75

*n* number of participants for the respective item, *m* mean, *SD* standard deviation

98% of parents (*n* = 107) and 96% of patients (*n* = 53), who answered the respective question, stated that they would “definitely agree” or “agree” to be treated on the IPAPAED again, if another inpatient stay was needed. The remaining participants stated that they would “rather agree” to be treated on the IPAPAED again. The willingness to be treated on the IPAPAED ward again was significantly correlated with appraisal of overall care (Spearman’s r for parents: *r* = .43, for patients: *r* = .52) and perceived quality of interprofessional collaboration (parents: *r* = .47, patients: *r* = .71) in both parents as well as patients (*p* < .001 for all).

### Free-text comments

Positive comments (*n* = 81) of parents mostly related to both well perceived interpersonal and communicative competencies like friendly personnel (*n* = 23, e.g. “the whole team was very friendly and helpful”), information sharing (*n* = 13, e.g. “very competent and transparent delivery of information”) and communication (*n* = 13, e.g. “helpful discussion during ward rounds with all medical students, nurses in training, physicians, and nurses”) as well as good quality of overall care (*n* = 21, e.g. “excellent care”). Twenty-seven comments just stated that everything was seen as positive.

Seventeen critical comments were given by parents, of those four were IPAPAED related namely the wish for more experienced doctors (*n* = 2, e.g. “maybe an experienced doctor should accompany students more frequently”), wish for more information about the program (*n* = 1) and for faster working on the ward (*n* = 1). All other comments concerned non-IPAPAED related items, most frequently the quality of rooms (*n* = 5) and food (*n* = 2) on the ward.

Forty patients gave positive comments, most frequently related to the friendliness of the personal (*n* = 10, e.g. “everyone was always very friendly”) and the overall care (*n* = 9, e.g. “great overall care”) as well as the communication (*n* = 4, e.g. “if I had questions everything was explained very well”).

Critical free-text comments of patients (*n* = 11) mainly targeted the quality of food (*n* = 6) but no IPAPAED-related aspects.

## Discussion

The overall aim of the IPAPAED ward is to enhance interprofessional learning and interprofessional collaboration to prepare doctors and nurses in training for their later work as members of interprofessional teams. The work presented here aims to evaluate both patients’ and parents’ perceptions of overall, medical and nursing care on a pediatric ITW. These patient perceptions of the care on the IPAPAED ward are an important factor regarding acceptance of such an educational intervention by parents, patients and health care professionals. The data presented here, to our knowledge, is the first assessment of an interprofessional education ward in a paediatric setting from a parental and paediatric patients‘perspective. The overall feedback by parents and patients of all age-groups alike was overwhelmingly positive – both in the quantitative evaluation as well as in the free-text comments. Parents showed a higher response rate than patients, this might be due to younger children not filling out or not being able to fill out in the evaluation.

Similar positive parental evaluation data have been reported for a monoprofessional paediatric CTW where medical students take care of paediatric patients and a similar perceived level of care compared to a normal paediatric ward was found [3]. However this work does not report paediatric patients’ assessments of their hospital stay in contrast to the study presented here.

Different studies have shown that patients’ evaluation results and standard of care were at least similar in both ITWs and conventional wards in adult medicine settings, mostly in orthopaedic wards, but also emergency, internal medicine, neurology, and geriatric settings [4–7, 10] and patient satisfaction was even higher on some ITW wards hinting at a probable halo effect of the interprofessional approach itself [4, 5, 8]. Patients especially commended the communication with and information they received by students, more individual attention by the interprofessional teams, and that they felt that students were very motivated and enthusiastic to work on the ITW [4–7, 9, 10]. Our free-text results demonstrate that both patients and parents on the IPAPAED ward appreciated the exact same characteristics in our nurses and physicians in training. Appreciation of both interpersonal and communicative competencies but also a professional level of care, as found in the limited sample of free-text patient and parent feedback, are reassuring. Maybe the best marker for the perceived high quality of care on the IPAPAED is that 96% of patients and 98% of parents stated that they would agree to be treated on the IPAPAED ward again if needed. These results are even better than for one monoprofessional internal medicine CEW that reported that 87.5% of patients agreed to be treated on the CEW again [18].

Being responsible for patient care for the first time motivated the participating nurses and physicians in training to deliver excellent care. The main difference to other senior students on the IPAPAED is that the interprofessional responsibility for the patients as a team is actively encouraged and expected. Traditional education on clinical wards depends on the willingness of nurse and physician supervisors to hand over responsibility to trainees and students and is far from standardized in Germany even though suggestions on standardizing at least the last year of medical school exist [19]. This active participation of nurses and physicians in training leads to a direct relationship of students with their patients which seems to be a pivotal factor for the success of both monoprofessional and interprofessional CEWs and is essential for patient-centered care but a halo effect of more structured mono- or interprofessional training cannot be excluded [3, 4, 20]. It might be that due to high motivation to perform and excellent communication within the interprofessional team as well as with patients and parents no treatment errors occurred on the IPAPAED so far – even with nurses and doctors just at the beginning of their careers.

Both nurse and physician trainees on the IPAPAED were equally valued in the evaluation and the interprofessional teamwork was praised. This hints at a high motivation to deliver the best care possible in both groups of trainees and that the teams are perceived as working together interprofessionally. The interprofessional setting itself has been attributed to higher patient satisfaction [8] and the same might hold true for the IPAPAED ward.

Nurses and physicians in training have a “safety net” of experienced registered nurses and certified physicians who are on the ward at all times during their rotation on the IPAPAED. These supervisors are an important part of their team and their presence, even if only in the background, and sometimes not in the same room, might be reassuring for patients as well [8]. The supervisors ensured that the IPAPAED team discussed the patients and their individual goals or changes to therapy before entering the patient rooms and gave input to formulate those, if needed. They helped to initiate the participation of patients and parents in the decision making process, if needed. So a specific care and treatment plan was formulated beforehand as it has been described before [8]. After the ward rounds in the patient room the team gathered again with the supervisors for a short reflection of the patient interaction to highlight positive aspects, identify improvable areas, and to discuss the individual patient’s resources from a nursing and medical point of view. This setting was chosen to establish a group culture of team work [21] and that all involved persons always had the same level of information regarding the individual patients.

Considering positive evaluation data from monoprofessional paediatric CTWs [3, 12] and ITWs in other fields of medicine than paediatrics [4–10], our data suggest that an ITW in paediatrics is well received by patients and their parents. The care that nurses and doctors in training deliver is perceived as excellent on the IPAPAED ward.

### Limitations and strengths

This study is limited by the lack of a control group of patients and parents. Standard patient feedback forms were available on the ward at all times but participation rates are traditionally low and feedback mainly concerns perceived low quality of the food and rooms. As the questionnaire employed for the IPAPAED ward is specifically aimed at assessment of interprofessional care and is longer than the standard questionnaire we chose not to employ it on the regular ward. All data reported are subjective impressions and no objective quality parameters (e.g. readmission rates) have been recorded. The specific focus on patient-centered care, motivated nurses and physicians in training and supervision by experienced registered nurses and certified doctors may contribute to the overall positive evaluation.

The strength of this study is that it is the first to report a paediatric patients’ evaluation of an ITW and shows that both patients and parents equally appreciate the care received on a paediatric ITW. This is encouraging for others who might be in the process of starting a paediatric IPW.

## Conclusions

From a parent as well as a patient perspective the nursing and medical care on a paediatric ITW is perceived as being excellent and no adverse opinions arose from either parents or patients regarding the care on the paediatric ITW. Both groups of trainees seem to contribute equally to the care in the view of parents and patients. Especially friendliness, quality of care, and communicative competencies are well received but the data stem from a limited sample of free-text commentaries. Our data support the implementation of ITWs even in the complex and specific setting of paediatrics.

## Data Availability

The datasets used and/or analyzed during the current study are available from the corresponding author on request.

## Abbreviations

CEW: Clinical education ward
IPAPAED: Interprofessional training ward in the setting of a general paediatric ward
ITW: Interprofessional training ward
n: number
SD: Standard deviation
